# Animal Hygiene Indexes in Relation to Big-Five Personality Traits of German Pig Farmers Evaluated by Self- and Other-Rating

**DOI:** 10.3389/fvets.2019.00379

**Published:** 2019-11-20

**Authors:** Susanne Döring, Nicole Geisthardt, Henrike Freitag, Iris Kobusch, Marc Boelhauve, Marcus Mergenthaler

**Affiliations:** Faculty of Agriculture, South Westphalia University of Applied Sciences, Soest, Germany

**Keywords:** pig farmer, biosecurity, animal hygiene, personality, advisory process

## Abstract

Improving biosecurity in intensive livestock production has become an increasingly challenging task. Often, animal hygiene measures are implemented at lower levels than recommended. Therefore, veterinarians and farm advisors look for new approaches to improve their advisory process with farmers. In the current study it has been hypothesized that German pig farmers' big-five measured personality traits might correlate with farms' biosecurity level expressed by a “continuous animal hygiene index” and a “technical animal hygiene index.” Hence, comprehensive data on the implementation of more than 100 hygiene measures were collected at farm level from a specific pilot sample of 42 pig farmers from a livestock intensive region in north-western Germany. In addition, big-five personality traits (BFI-S) were measured by self- and other-rating. Inter-rater reliabilities for personality traits indicated expected positive correlations apart from agreeableness (*r*_S_ = −0.101). Regarding the self-rating, neuroticism was valued lowest ($\bar{\text{x}}$ = 3.88 ± 1.18) and conscientiousness highest ($\bar{\text{x}}$ = 5.68 ± 0.70). The animal hygiene indexes revealed medium biosecurity levels on the participating farms. Piglet breeders had a significantly higher value for the “continuous animal hygiene index” ($\bar{\text{x}}$ = 63.00 ± 9.91%). Personality traits conscientiousness and openness showed correlations with the continuous and the technical animal hygiene index. Depending on the production systems as well as the rating perspectives, correlations varied. For one of the personality traits playing a direct role in social interaction—extraversion—the advisory process might function as a mediating factor. The current results show that clustering of single hygiene measures into indexes in the evaluation of pig farms' biosecurity level might have advantages. The preliminary results from this study should be validated in larger, more representative samples. Furthermore, structured and systematic consideration of personality traits of farmers adds an additional aspect to include individuality of farmers more systematically in complex advisory processes. Interaction of personality traits with characteristics of the advisory process should be further researched and should be included in a much broader socio-political understanding of what is involved in changing practices.

## Introduction

Animal hygiene has become a “mainstream prerequisite for an ethically accepted and sustainable production of food from animals” (1). Nevertheless, measures to enhance animal hygiene are often implemented at levels lower than recommended. Not least of all, animal performance could increase, if animal health and hygiene were enhanced (2). Therefore, there has been a recent increase in research aimed at a valid evaluation of current biosecurity levels and practicing biosecurity measures on livestock farms [e.g., (3–6)]. But even when biosecurity levels were regularly highlighted as strongly in need of improvement, reasons for low implementation were often unclear.

Zoonotic diseases, which can affect food animal populations as well as human health, still play a major role (7). Especially intensive pig livestock regions, such as the north-western part of Germany, are susceptible to epidemic outbreaks. Concerning the African swine fever, for example, there is a high risk of dissemination from Eastern Europe through food leftovers, feral pigs as well as pig livestock imported to Germany. Dissemination depends on biosecurity levels of pig farms (8), among others. Whereas, the African swine fever does not harm humans, pig-transmitted Methicillin-resistant Staphylococcus aureus (MRSA) colonization of German farm workers has been proven in several studies [e.g., (9, 10)]. So, the level of endemic infection of pig herds is relevant concerning hospital-transmitted MRSA infections (11). Further examples given are Salmonella infections, whose harm is not limited to pig health. Nowadays, people still become ill by food-borne salmonellosis infections (12–14). They also cause the most deaths regarding foods of animal origin in Germany (15). Therefore, the implementation of biosecurity measures on livestock farms as a preventive approach has become an increasingly important task.

Here the question arises of how implementation of measures can be enhanced at the farm level. Implementation deficits have been identified in several studies [e.g., (5, 16, 17)]. Research clearly showed that pure knowledge about useful on-farm measures with concern to livestock husbandry is lost before their implementation (18, 19). It was also shown that biosecurity measures were considered derogatorily by many farmers (20). Distinctly, perceptions and attitudes toward the implementation of single hygienic measures at any rate, recommended by science and mediated by veterinarians or farm advisors, have been a worldwide problem for years, as discussed by Racicot et al. (4). Hence, veterinarians and farm advisors look for new approaches to overcome the lack of implementation of measures. Moreover, these persons are the most important ones, who can highly impact on farmers' decision making and attitudinal behavior (16, 21–26). Therefore, communication and understanding between all agents is necessary (16).

Veterinarians and farm advisors need science's support to get access to valid and feasible tools, being applicable during farm visits. Indeed, veterinary epidemiology in combination with social sciences maintains a multidisciplinary approach. It is difficult to let results intertwine (24). Obstacles were attributed to researchers' specializations. Relevant interdisciplinary cooperation between veterinary and social sciences is often still missing. Following this scientific background, the most important and difficult tasks are still prospectively, (1) to be able to define the origin of farmers' general understanding and decision making behavior with regards to farm operating strategies and (2) to meet the challenge of deriving action strategies for veterinarian personnel and farm advisors.

In recent years there have been different approaches to analyze, especially psychological, motivational as well as social factors explaining and predicting farmers' behavior in relation to veterinary epidemiology as well as infectious diseases among farm animals (19, 21, 23, 27). Additionally, increasing research is available in which farmers' personality traits were assessed in relation to non-epidemic as well as health topics. Reliable predictors of their behavior could be identified (28–30). Thereby, the five-factor personality model or rather the big-five model, as a method originated in the “psycho-lexical-tradition” (31, 32) in combination with the “differentiated and clinical tradition of the personality research” (33, 34) was implemented successfully several times on different farmers [e.g., (35)]. Regarding farms' disease control as well as farms' biosecurity compliance, researchers found that several personality traits are highly correlated for the assessed dependent variables and measures (4, 36, 37). Because of these recently obtained and demonstrably useful signs on the applicability of the big-five personality model on cattle as well as poultry farmers, this model was chosen in the current study with intensive pig farms. Here, it has been generally hypothesized that farmers' big-five measured personality traits might have significant impact on pig farms' biosecurity levels. Hypothetically, information on farmers' personalities could support veterinarians and farm advisors to develop more tailored advisory processes and strategies.

**Figure 1:**
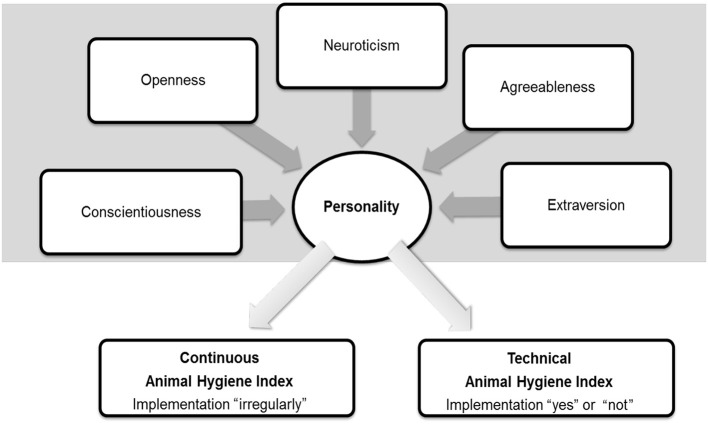
Big-five model with the five personality traits aligning the “continuous animal hygiene index” and the “technical animal hygiene index” with their frequencies of implementation of measures.

Therefore, the first aim of the current study was to test the big-five model “BFI-S” (38) for reliability by self- and other-rating, which has not been done in animal health-related studies before. The sample comprised 42 German intensive pig livestock farmers. They were part of a more comprehensive three-year research project. The second aim was appraising the implementation level of biosecurity measures. For this purpose, a survey of farmers participating in the project was conducted by two researchers during a farm visit. The third aim of the present study was to analyze the impact of big-five measured personality traits on the farms' biosecurity levels. Therefore, it was hypothesized that different measures occurring on farms were influenced, to various extents, by different personality traits (Figure 1).

## Data and Methods

### Project Design

Farmers participating in the current study joined the three-year project “Preventive hygienic consulting” which ran from 2014 to 2017. The project was for improving animal hygiene in intensive, conventional pig production in a livestock-intensive region in the state of North Rhine-Westphalia in north-western Germany. The project included workshops on biosecurity measures, possibility for on-farm research trials, farm individual biosecurity consulting and the possibility to engage a professional pest-control operator at subsidized fees. Farmers were suggested by regional advisors from different organizations and veterinarians to participate in two project information workshops in October 2013. Due to the pilot character of the present study, it was seen as essential to work within established on-farm research structures and build on trust relationships with these farmers. All farmers were pig producers at different levels in the production chain. Famers in our sample were from three production systems of “breeding sow keepers” (*N* = 8), “piglet breeders” (*N* = 10), and “fattening pig keepers” (*N* = 24). The three different production systems chosen were classified according to common conditions as follows:

1. BSK: Breeding sow keepers (sows and piglets till 8 kg body weight)
2. PB: Piglet breeders (piglets from 8 to 25 kg body weight)
3. FPK: Fattening pig keepers (pigs from 25 kg body weight to slaughter weight)

As shown in Table 1, some farms were comprised of two or all three production systems with every possible combination. Thus, for the current analyses, farms were classified according to the production system self-selected by farmers. Data were based on stables and partly accounted for overall farm hygiene. On average, breeding sow keepers kept 438 ± 125 sows, piglet breeders 1,265 ± 673 piglets and fattening pig keepers 2,262 ± 1,434 pigs.

**Table 1:** Number of kept animals disaggregated by production systems.

	**Sows**	**Piglets**	**Fattening pigs**
	$\bar{\text{x}}$ ± $\tilde{\text{x}}$	$\bar{\text{x}}$ ± $\tilde{\text{x}}$	$\bar{\text{x}}$ ± $\tilde{\text{x}}$
Breeding sow keepers (BSK)	438 ± 125 (8^*^)	1,253 ± 799 (6)	640 ± 792 (2)
Piglet breeders (PK)	360 ± 310 (5)	1,265 ± 673 (10)	1,288 ± 165 (4)
Fattening pig keepers (FPK)	84 (1)	643 ± 367 (3)	2,262 ± 1,434 (24)

* *Number of farms in brackets*.

Participation in the research project was voluntary. Project data for this study was collected by the help of two questionnaires within face-to-face interviews. The first was the “intensive farm questionnaire,” which was implemented to build animal hygiene indexes. Furthermore, overall project evaluation data as well as big-five personality traits assessed by self- and other-rating were collected by the “concluding farm questionnaire.” Data was collected in such a way that data from the two different surveys could be linked for each farm.

### Intensive Farm Questionnaire

A comprehensive questionnaire was developed and implemented on farms from January to April 2014 in order to conduct a detailed overall evaluation of the hygienic situations. The survey was done face-to-face with the respective farmers by two researchers during a farm visit. A specific questionnaire was developed for each different production system containing specific items referring to the production system in question, as well as containing an equal main part, which was divided into six farm compartments (Table 2). Biological performance indicators were queried as well but not included in the current study. Most of the items were polar questions with predefined reply classes. Depending on the type of question, each reply class was named or only the polar points were named. Additionally, some open questions were asked and constitute additional items.

**Table 2:** Farm compartments comprised by the “intensive farm questionnaire” for the production systems breeding sow keepers, piglet breeders, and fattening pig keepers.

**Farm compartment**	
1	Stable
2	Farm organization
3	Farm hygiene
4	Stable climate
5	Health prophylaxis
6	Biological performances

### Animal Hygiene Index

As measures are implemented on farms to varying degrees, the items of the “intensive farm questionnaire” were firstly divided by their frequency of measure implementation. Following this, there were two kinds of frequencies. The first concerns whether or not measures are continuously implemented or, if they are generally conducted or not. The latter were related to structural conditions and considered as technical measures. Thus, a detailed definition of the “continuous animal hygiene index” and the technical hygiene index is presented in the following two sections.

### Continuous Animal Hygiene Index (CAHI)

The “continuous animal hygiene index” includes operational measures conducted at different frequencies, even if they should be carried out regularly. Regularly means in relation to the intended objective (once or more times a day, once after emptying the stable, etcetera). These measures relate to the farmers' operational behavior and decisions. Examples of measures considered in this index are listed in Table 3.

**Table 3:** Number of items according to the production systems and content examples of items related to the “continuous animal hygiene index” (CAHI).

**Production system**	**Number of items**	**Content of items**				
Breeding sow keepers (BSK) (*N* = 8)	80	Take on/off farm-owned protection clothes	Cleaning/disinfecting protecting shoes	Cleaning appliances/water/feed pipelines	Conducting deworming	Dissection of pigs with unknown cause of death
Piglet breeders (PB) (*N* = 10)	67					
Fattening pig keepers (FPK) (*N* = 24)	72					
				**Answer options**		
		Always/ mostly/ sometimes/ never	Always/ mostly/ sometimes/ never	After every trial/ yearly/ sometimes/ never	Yes/ partly/ no	Always/ partly/ rarely/ never

### Technical Animal Hygiene Index (TAHI)

The “technical animal hygiene index” relates to measures of structural implementation and in relation to technical conditions. As such, these measures relate to farmers' strategic behavior and decision-making. Examples of measures considered in this index are provided in Table 4.

**Table 4:** Number of items according to the production systems and content examples of items related to the “technical animal hygiene index” (TAHI).

**Production system**	**Number of items**	**Content of items**				
Breeding sow keepers (BSK) (*N* = 8)	38	General structural state of the stable	Conducting the “all in - all out” and “black—white' system	Providing a changing room with shower and visitor protocol	Storage of feed and litter saved from sun and animals	Conduction of water samples analyses and water disinfection
Piglet breeders (PB) (*N* = 10)	34					
Fattening pig keepers (FPK) (*N* = 24)	38					
				**Answer options**		
		Very good/ good/ in need of renovation/ very in need of renovation	Yes/ no	Yes/ no	Yes/ partly/ no	Yearly/ if required/ never

### Animal Hygiene Index Calculation

**Figure 2:**
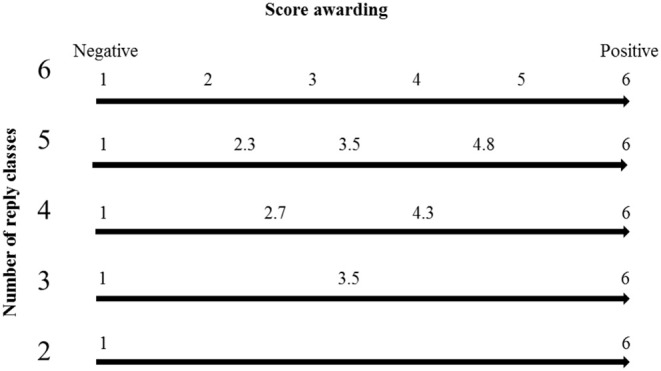
Score awarding for the animal hygiene indexes (CAHI, TAHI) for two to six reply classes.

The animal hygiene indexes (CAHI and TAHI) were calculated as two separate indexes for every single farm. Thereby, all items were assigned either to be included in the CAHI or in the TAHI calculation. Secondly, the points given for every question were divided proportionally to the number of reply classes as shown in Figure 2. High levels of implementation correspond to six points and low levels to one point. The numbers of reply classes reflect the implementation of measures (i.e., the frequency of implementation of measures or if specific structural conditions are present or not). If no hygiene measures were implemented, this was evaluated by one point. Further open answers were coded to reply classes, too. These reply classes were defined ex-post to the survey during data analysis. Examples include the type of washing equipment for shoes or the strategy to reduce flies. Afterwards, all items were weighted based on their relevance with regard to hygiene levels by factor multiplication. The factors run from 1 (low factor loading) over 2 (medium factor loading) to 3 (high factor loading). The ranking was carried out by the three researchers conducting this study, all having a pig production advisory background. Altogether, the CAHI and the TAHI each represent the sum of the achieved question points multiplied with the determined factors for every single farm, always in relation to the maximal reachable sum of points over all items, as the following formula shows.

(1) $$\begin{array}{r} V_{\frac{C}{T}}\left(j\right)=1/z\overset{q}{\underset{i=1}{\sum }}\left(x_{ij}*y_{i}\right)*100\% \end{array}$$

V_C_ = Value of “continuous animal hygiene index” (CAHI)

V_T_ = Value of “technical animal hygiene index” (TAHI)

i = Items

j = Farmer

q = Number of items

x = Scored number of reply classes

y = Factor-loading

z = Maximal reachable sum of points from all items.

### Concluding Farm Questionnaire

The “concluding farm questionnaire” was designed for a final project evaluation. Therefore, farmers were visited again during December 2016 to February 2017. This questionnaire evaluated the overall project at the end, i.e., if the project measures provided resulted in increased hygienic conditions on farms; if the farmers' hygienic awareness has changed. Both aims were not the focus of the current study. Parts of the project evaluation results have been published by Wildraut et al. (39) and Hecker et al. (40). The big-five assessment was included in the “concluding farm questionnaire” as explained in the following section.

### Big-Five Personality (BFI-S) Assessment

For the personality trait assessment, the “BFI-S” from Schupp and Gerlitz (38) was chosen. The farmers valued their approval or disapproval for the items (for items' content, see the second column of Table 5; items were linguistically shortened) on terminal seven-pointed Likert scales (“does not apply at all” to “fully applies”). For the self-ratings, all items started with “I am somebody, who….” For the other-rating, the items started with “The farmer is somebody, who….” For the other-rating, the person was an affiliated person who knew the farmer well. As the study was done in a context with family farms, the farmers' wives were mostly chosen by the farmers as the person doing the other-rating. Reverse scaled items were rescaled before data analysis was conducted, so that all items were expressed positively: High scale values mean high approval (see column “item-polarity” in Table 5). Results were always presented for the self- and other-rating.

**Table 5:** Means, standard deviation, medians, inter-rater-, and inter-item correlations (*r*_S_, ^*^p ≤ 0.05, ^**^p ≤ 0.01) of the big-five traits based on the BFI-S for intensive pig keepers (*N* = 42).

**Trait**	**Item**	**Item-no. (Item-polarity)**	**Means** **±** **standard deviation (Median)**		**Differences of the means**	**Inter-item correlations**			**Interrater-correlation**
			**Self**	**Other**		**Items**	**Self**	**Other**	
Extraversion	communicative, talkative	1 (+)	5.38 ± 0.96 (6.0)	5.95 ± 1.08 (6.0)	−0.57 ± 1.15	1↔2	0.443^**^	0.205	0.397^**^
	reserved	2 (–)	3.36 ± 1.41 (4.0)	4.45 ± 1.85 (4.0)	−0.10 ± 2.02	2↔3	0.411^**^	0.452^**^	0.268
	outgoing, sociably	3 (+)	5.17 ± 1.17 (5.0)	5.95 ± 1.13 (6.0)	−0.79 ± 1.14	1↔3	0.452^**^	0.442^**^	0.530^**^
	**Mean**		**4.97** **±** **0.93 (5,0)**	**5.45** **±** **1.01 (5.3)**	**−0.48** **±** **0.92**				0.515^**^
Conscientiousness	working thoroughly	1 (+)	5.57 ± 0.86 (6.0)	6.24 ± 0.93 (6.0)	−0.67 ± 0.93	1↔2	0.520^**^	0.675^**^	0.393^**^
	effective and efficient	2 (+)	5.81 ± 0.80 (6.0)	6.14 ± 1.20 (6.5)	−0.33 ± 1.20	2↔3	0.073	0.260	0.351^*^
	rather lazy	3 (–)	5.67 ± 1.39 (6.0)	6.43 ± 1.42 (7.0)	−0.76 ± 1.59	1↔3	0.225	0.374^*^	0.139
	**Mean**		**5.68** **±** **0.70 (5,7)**	**6.27** **±** **0.93 (6.5)**	**−0.59** **±** **0.95**				**0.212**
Neuroticism	getting nervous easily	1 (+)	3.69 ± 1.62 (4.0)	3.52 ± 1.80 (3.0)	0.17 ± 1.67	1↔2	0.325^*^	0.644^**^	0.518^**^
	relaxed, doing well with stress	2 (–)	3.55 ± 1.44 (3.5)	3.29 ± 1.73 (3.0)	0.26 ± 2.10	2↔3	0.112	0.019	0.179
	worrying often	3 (+)	4.40 ± 1.77 (4.5)	4.43 ± 1.71 (4.5)	−0.02 ± 1.92	1↔3	0.477^**^	0.194	0.326^*^
	**Mean**		**3.88** **±** **1.18 (3,8)**	**3.75** **±** **1.27 (3.7)**	**0.14** **±** **1.21**				0.440^**^
Openness	appreciating artistic experiences	1 (+)	3.40 ± 1.56 (4.0)	3.88 ± 1.66 (4.0)	−0.48 ± 1.74	1↔2	−0.160	0.246	0.420^**^
	vivid phantasy, having imagination	2 (+)	4.57 ± 1.42 (5.0)	4.38 ± 1.55 (4.0)	0.19 ± 1.86	2↔3	0.153	0.602^**^	0.170
	ingenious, introducing new ideas	3 (+)	5.02 ± 1.32 (5.0)	5.60 ± 1.08 (6.0)	−0.57 ± 1.58	1↔3	−0.032	0.331^*^	−0.066
	**Mean**		**4,33** **±** **0,76 (4,3)**	**4.62** **±** **1.10 (4.7)**	**−0.29** **±** **1.17**				**0.216**
Agreeableness	considering and friendly	1 (+)	5.71 ± 0.86 (6.0)	5.86 ± 1.07 (6.0)	−0.14 ± 1.56	1↔2	0.362^*^	0.551^**^	−0.275
	sometimes rude to others	2 (–)	5.17 ± 1.40 (5.0)	5.55 ± 1.58 (6.0)	−0.38 ± 2.06	2↔3	0.180	0.317^*^	0.113
	forgiving	3 (+)	5.57 ± 1.09 (6.0)	5.74 ± 1.38 (6.0)	−0.17 ± 1.78	1↔3	0.124	0.280	0.178
	**Mean**		**5,48** **±** **0,76 (5,7)**	**5.71** **±** **1.02 (5.8)**	**−0.23** **±** **1.39**				**−0.101**

### Statistical Analyses

Data entry was done with Microsoft Excel 2010 and statistical analyses with IBM SPSS Statistics 25. All data were first analyzed descriptively. Afterwards, data were tested for homogeneity of variances and normal distribution using the Levene and Kolmogorov-Smirnov procedures, respectively. Variance homogeneity was given for all BFI-S personality traits except neuroticism. Additionally, sample sizes for the farmers of the three different production systems differed widely. Hence, differences between personality traits and production systems were analyzed by the Hochberg GT2 procedure (α = 0.05) after using the univariate ANOVA.

Differences of the big-five traits (dependent variable) between production system (independent variable) were analyzed answering the question if personalities of farmers differ between production systems.

Concerning the continuous (CAHI) and technical animal hygiene indexes (TAHI), variances were homogenous and data were distributed normally for the technical index (TAHI). Regarding the different sample size of the production systems and the robustness of the Hochberg GT2 procedure (α = 0.05) against non-normal distributed data, this test was chosen for analyzing the differences of the indexes (dependent variable) between pig production systems (independent variable), answering the question if implementation of hygiene measures differs between production systems.

Inter-rater and inter-item correlations of the big-five personality traits were calculated. Inter-rater correlation refers to the correlation between self- and other-rating. High correlations indicate a high reliability of the measured item. Inter-item correlations refer to correlations between the three single items of each personality trait. High correlations indicate high internal construct validity of the respective personality traits. Since items were measured as ordinate variables, the Spearman rank-correlation (r_S_) was used. Together, these analyses were implemented to test for the consistency of the big-five personality model in the context of this study. For comparison of the continuous (CAHI) and “technical animal hygiene index” (TAHI) as metric parameters, the Pearson correlation was calculated (r_P_). Correlations of the big-five personality traits by self- and other-rating with the “continuous animal hygiene index” (CAHI) and the “technical animal hygiene index” (TAHI) were analyzed by the Spearman rank-correlation (r_S_). These analyses answer the key question of the study whether personality traits are correlated with the implementation level of hygiene measures. The reference unit for all analyses was the farms (always *N* = 42).

## Results

### Inter-rater and Inter-item Correlations

Regarding the self-ratings with an average value of 5.68 ± 0.11 for conscientiousness, this trait was valued highest, followed by agreeableness ($\bar{\text{x}}$ = 5.48 ± 0.12, Table 5) and extraversion ($\bar{\text{x}}$ = 4.79 ± 0.14). Openness was valued with 4.33 ± 0.12 on average and neuroticism lowest ($\bar{\text{x}}$ = 3.88 ± 0.18). The highest mean difference between self- and other-rating was 0.59 ± 0.95 data points, which was related to conscientiousness. The other-rating always resulted in higher valued items.

Regarding the self-rating, inter-item correlations were significant concerning extraversion as well as particularly concerning conscientiousness, neuroticism as well as agreeableness. Regarding other-rating, correlation was found between two pairs of items in relation to extraversion, conscientiousness, openness, and agreeableness, whilst only one pair of items was significant for neuroticism. Significant inter-rater correlations were found for some of the personality traits (Table 5).

### Differences Between the Production Systems for the BFI-S Traits

**Figure 3:**
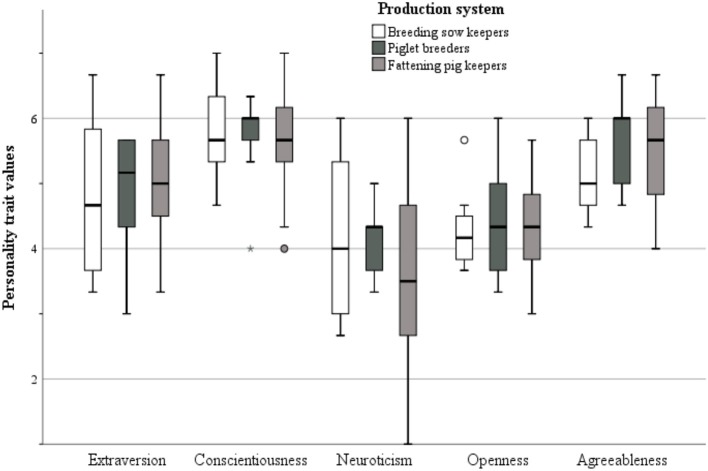
Box-and-whisker plot of personality trait values according to the pig production system: breeding sow keeper (*N* = 8), piglet breeders (*N* = 10) and fattening pig keepers (*N* = 24).

Differences between all five of the personality traits were not significant in relation to the three production systems “breeding sow keepers” (BSK), “piglet breeders” (PB), and “fattening pig keepers” (FPK) (extraversion: *F* = 0.267, *p* = 0.767; conscientiousness: *F* = 0.140, *p* = 0.870; neuroticism: *F* = 0.702, *p* = 0.502; openness: *F* = 0.110, *p* = 0.896; agreeableness: *F* = 1.355, *p* = 0.270). As a tendency, piglet breeders valued themselves highest in all traits (Figure 3). In this production type, conscientiousness as well as agreeableness was valued highest with always a median of six. Lowest values were chosen for openness ($\tilde{\text{x}}$ = 4) and neuroticism ($\tilde{\text{x}}$ = 4). Breeding sow keepers valued themselves highest in regards to conscientiousness ($\tilde{\text{x}}$ = 6) and lowest regarding neuroticism ($\tilde{\text{x}}$ = 4) and openness ($\tilde{\text{x}}$ = 4), when comparing the different traits within this production system. Further, the fattening pig breeders had the widest standard deviation for neuroticism with additionally the lowest median value ($\tilde{\text{x}}$ = 4). They rated themselves highest with conscientiousness and agreeableness (always $\tilde{\text{x}}$ = 6).

### Differences Between the Production Systems for the CAHI and TAHI

**Figure 4:**
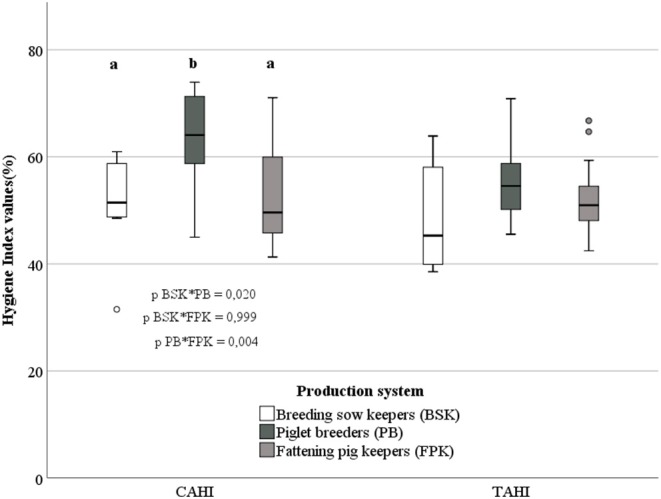
Box-and-whisker plot of continuous and “technical animal hygiene index” values (%) according to the pig production systems. Different letters indicate significant differences (ANOVA and Hochberg GT2 procedure; α = 0.05); *p*-values of pairwise comparisons are given below the box-and-whisker plots.

The value for the CAHI was significantly higher for piglet breeders in comparison with breeding sow keepers and fattening pig keepers (Figure 4). Breeding sow keepers and fattening pig keepers did not differ significantly (*p* = 0.999) with regard to the CAHI. Regarding the TAHI, no significant differences occurred between the three systems (*F* = 1.837, *p* = 0.173). The Pearson correlation between the indexes revealed a significant positive but medium result concerning fattening pig keepers (*r*_P_ = 0.639, *p* = 0.01). Further medium non-significant correlation occurred for breeding sow keepers (*r*_P_ = 0.607) and low negative correlation for piglet breeders (*r*_P_ = −0.188), which was not significant either. Altogether, the indexes correlated significantly but with moderate value (*r*_P_ = 0.451, *p* = 0.01).

### Big-Five Personality Traits in Correlation With the CAHI and TAHI

Few significant correlations were found between the hygiene indexes and personality traits (Table 6). For example, significant correlations occurred with the fattening pig breeders (FPB), who achieved higher index values for both indexes, when conscientiousness was valued higher regarding the self-rating (CAHI: *r*_S_ = 0.453, *p* = 0.05; TAHI: *r*_S_ = 0.419, *p* = 0.05). With regards to breeding sow keepers (BSK), openness is significantly and highly positively correlated with the TAHI in respect to the self-rating (*r*_S_ = 0.764, *p* = 0.05). For the piglet breeders' (PB) other-rating, there is a significant negative correlation for extraversion with the TAHI (*r*_S_ = −0.691, *p* = 0.05).

**Table 6:** Spearman correlations (*r*_S_, p* ≤ 0.05) of the big-five personality traits (BFI-S) by self- and other-rating with the “continuous animal hygiene index” and the “technical animal hygiene index” according to the production systems.

**Trait**	**Continuous animal hygiene index**					
	**Self-rating**			**Other-rating**		
	**BSK**	**PB**	**FPK**	**BSK**	**PB**	**FPK**
Extraversion	−0.455	0.624	0.251	0.072	0.265	−0.132
Conscientiousness	−0.265	0.519	0.453^*^	0.049	0.304	−0.010
Neuroticism	0.386	0.120	−0.094	0.570	−0.161	−0.248
Openness	0.497	0.530	0.140	0.220	0.332	−0.183
Agreeableness	0.170	0.000	−0.236	0.563	−0.086	0.202
	**Technical animal hygiene index**					
Extraversion	−0.467	−0.333	0.097	−0.012	−0.691^*^	−0.177
Conscientiousness	−0.072	0.337	0.419^*^	−0.195	0.076	0.126
Neuroticism	0.169	−0.031	−0.060	0.218	0.019	0.118
Openness	0.764^*^	−0.439	0.389	0.293	−0.049	−0.188
Agreeableness	0.485	0.058	−0.022	0.240	0.295	−0.070

*BSK, Breeding sow keepers (N = 8); PB, Piglet breeders (N = 10); FPK, Fattening pig keepers (N = 24); All, All farmers (N = 42)*.

## Discussion

### Inter-rater and Inter-item Correlations of Big-Five Personality Items and Traits

Inter-rater reliabilities as well as inter-item reliabilities indicated mostly expected signs of the correlation coefficients. Inter-item correlations were predominantly in medium range with partially significant results. In general, lower correlations occurred within self-ratings. The results give first indications about the appropriateness of single items for evaluating personality traits when using the BFI-S among pig farmers. In addition, for single items as well as personality traits wide standard deviations were observed, especially for other-ratings. This is an indication that farmers' personality differences can be evaluated with the BFI-S and there are no stereotypical valuations.

Certain problems can be detected to measure the trait openness. For “appreciating artistic experiences” as well as “ingenious, introducing new ideas” from the openness trait, low negative correlations were found (*r*_S_ = −0.160 and −0.032) in the self-ratings. With the item “appreciating artistic experiences,” Lang et al. (41) also identified inconsistent correlations in the self-ratings while testing for item-reliability. Perhaps this trait should be rephrased or even exchanged by another item in future self-ratings. Openness should possibly be rather assessed by other-ratings as inter-item correlations where considerably higher for other-ratings. Additionally, with conscientiousness as well as agreeableness, higher inter-item correlations regarding the other-rating could be identified.

The low negative yet non-significant total inter-rater correlations for agreeableness were caused by low inter-rater correlations for single items. In particular, the item “considering and friendly with others” did not correlate between self- and other-rating. Similar findings on agreeableness were found by Conelly and Ones (42), who did a meta-analytical study on observers' accuracy. This study found that for traits which were high in being evaluative—as agreeableness—personal closeness does not lead to higher inter-rater reliability. With agreeableness, inter-item correlations were higher for other-ratings than for self-ratings. This gives an indication that agreeableness should rather be measured by other-ratings in future studies.

Total inter-rater correlations appeared significant for extraversion and neuroticism. However, Vazire (43) found that neuroticism is assessed more reliably by other-rating. Indeed, this was not found in the present study. Here inter-item-reliability was good for self- and other-rating, and inter-rater reliability was good (*r*_S_ = 0.440, *p* = 0.01). A review on work performance and personality discovered that neuroticism's prediction ability was not at all satisfying (44). The authors concluded that broader constructed items would make this trait more valid. With “resiliency” vs. “internalizing negative emotionality” as opposed to neuroticism, the “Questionnaire Big Six Scale” could meet these requirements with greater validity.

Due to medium satisfaction with the reliability of the BFI-S, the implementation of a more specialized personality trait questionnaire, such as the “Questionnaire Big Six Scale,” could lead to more reliable results. In that model, “honesty-humility” is separated from agreeableness into an independent sixth personality trait. Additionally, different subsets of the original 120-item survey (45) are available and already tested for item validity (46, 47). For practical reasons, it might be difficult to use longer survey tools.

Overall, the results of the present study give a first indication that the BFI-S model could be a valid tool to measure pig farmers' personality traits. Yet, as the focus of this study has been much broader than testing the validity of the BFI-S model and as the sample size has been limited, more specific studies are required for validation of the BFI-S among pig farmers. Special focus should be given on self- and other-ratings as the present study gives indication that they differ in their reliability depending on the type of personality trait to be evaluated. Openness and agreeableness especially might be evaluated more consistently by other-ratings. This would be a challenge for many empirical studies as data on self-ratings can be collected much easier than data on other-ratings.

### The CAHI and the TAHI

The hygiene standard for pig livestock in Germany is at a medium level as data from the current study show. This applies to the main production systems breeding sow keepers, piglet breeders as well as fattening pig keepers and in relation to continuous (CAHI) and more structural (TAHI) biosecurity measures. With the highest values for the CAHI being 74% (PB; 71% = FPK, 61% = BSK) and for the TAHI being 71% (PB; 67% = FPK, 64% = BSK), results allow the conclusion that an increase of the farms' hygiene level is not only possible but also strongly recommended. Our results are in a similar range as results from Backhans et al. (5). These authors estimated biosecurity levels for farrow-to-finish herd farmers having reached 59–68 points on an average of a possible 100 for hygiene measure implementation.

Piglet breeders had the highest animal hygiene indexes, which could indicate increased awareness of hygienic practices and biosecurity during the very sensitive rearing period where piglets are prone to infectious diseases. The CAHI lead to measures, such as cleaning and disinfection procedures, being implemented more often. These measures lead to better pigs and piglet health (2, 28).

The CAHI seemed to be more important for sow keepers in comparison to fattening pig keepers. This suggests that sow keepers were more aware of the negative consequences when measures are not implemented than fattening pig keepers. Fertilization, birth and rearing periods are sensitive periods, which critically decide about the farms' economic success, too.

Additionally, further analyses have to be done to evaluate in which compartments the greatest deficits and potentials for improvement occurred to specifically target these farm-designed aspects. This should be done separately for every production system. On the other hand, it was difficult to attempt a total and detailed evaluation of the biosecurity level of the pig farms. Hygiene comprises many fields with its measurement of hygiene epidemics as well as hygiene within stable surfaces, water, air and feed [production, storage and its usage; (3)]. Still, the applied “intensive farm questionnaire” with at least 100 queried measures has included many important categories regarding important hygienic measures.

All items were evaluated by three agricultural researchers. This procedure was used for weighting the importance of single items. As such, it should be obvious that “Are further livestock animals kept at this location?” and “Is there a separating area for ill pigs within the stable?” have different weighting factors. The factor 1 (low factor loading) was chosen for the first and factor 3 (high factor loading) for the latter example item. An example for medium factor loading (i.e., factor 2) is “How long does the stable remain empty after cleaning and/or disinfection until its occupancy?” To conclude, clustering of single hygiene measures into indexes offers advantages in the evaluation of pig livestock farm situations.

### Big Five Personality Traits and the CAHI and the TAHI

Consideration of pig livestock farmers' personality traits opens new avenues of inquiry to include individuality of farmers more systematically in consulting processes of improving biosecurity in livestock production. The present results from the big-five models gave only weak hints as being related to biosecurity levels as measured by the two index values (CAHI, TAHI). In that way, results showed that conscientiousness and openness had significant impact concerning the CAHI as well as the TAHI. However, this appeared only for fattening pig breeders in respect to both indexes related to conscientiousness. Studies on work performance generally showed significant positive influence of conscientiousness from previous years [reviewed by (48)].

Breeding sow keepers achieved significantly higher values concerning the TAHI if openness was valued higher by self-rating procedures. Consequently, more open sow-keeping farmers tend to have more success in technical measures. This occurrence may be due to a greater inclination toward new techniques and a more digitalized workflow (e.g., from the current questionnaire: “Is a sensor-controlled feed trough implemented?”). Research showed for example that knowledge led farmers to use estrus detection techniques more often with dairy cattle (17). It can be concluded that more open farmers tend to have more interest in increasing their knowledge in this field. The result suggests that veterinarians and farm advisors have to develop strategies to specifically target farmers with lower levels in conscientiousness and openness.

Extraversion had a negative influence on the TAHI in respect to the other-rating with piglet breeders. This personality trait playing a direct role in social interaction in the advisory process might function as a mediating factor (49, 50). This research gap should be addressed in future studies. If these results could be further confirmed, knowledge of improved consulting processes should be transferred to veterinarians as well as farm advisors to meet requirements for successful consulting processes.

Advisory agents should significantly increase their efforts for understanding factors which lead farmer' decisions and behavior. First, it can be emphasized that understanding farmers' decision making and attitudinal behavior are worldwide obstacles to the common urgency of enhancing the biosecurity levels of livestock farms. Secondly, it can be considered that consideration of farmers' personality could improve biosecurity measures and animal health.

## Limitations of the Study

The present study aimed to analyze if the implementation of hygiene measures correlates with personality traits of intensive pig famers. The sample size of *N* = 42 can be considered limited and is not representative in a statistical sense. Collection of high-quality data about on-farm implementation of hygiene measures as well as data about personality traits require a trust-relation with farmers in the research process. Therefore, a certain tradeoff can be assumed between sample size and data quality. The results of the present study should not be generalized. Instead, they should be considered as a starting point for broader, more comprehensive studies about the link between implementation of hygiene measures in pig farming and the decision-makers in this process.

Moreover, a multidisciplinary approach should be employed to acquire valid and applicable recommendations for veterinarians and farm advisors, including psychologists, social researchers as well as animal scientists. A broader analytical perspective should also include a socio-political understanding of what is involved in changing practices. Hence, it becomes clear that the inclusion of big-five personality traits constitute only a partial explanation of the implementation of biosecurity measures, which has to be complemented by other factors (51, 52).

## Conclusion

For prevention of zoonotic disease spreading from German pig livestock farms, increased animal hygiene is essential. The underlying hypothesis for the present study was that intensive pig farms' biosecurity levels were influenced by farmers' personalities. As a conclusion from the present data, personality could be considered in advisory processes as one aspect among many. The results should be validated in larger, more representative samples with adapted survey tools. Moreover, future research should be done with respect to a multidisciplinary approach to include multifactorial-caused decision making and attitudinal behavior of livestock farmers.

## Data Availability Statement

The datasets generated for this study are available on request to the corresponding author.

## Ethics Statement

Local/national legislation does not require written approval of survey respondents. Verbal consent was acquired at the beginning of each interview. Approval of an ethic committee is not required for the study type.

## Author Contributions

All authors listed have made a substantial, direct and intellectual contribution to the work, and approved it for publication.

### Conflict of Interest

The authors declare that the research was conducted in the absence of any commercial or financial relationships that could be construed as a potential conflict of interest.
